# Amyand's hernia in a 2-month-old infant: The first case report from Sudan

**DOI:** 10.1016/j.ijscr.2024.110114

**Published:** 2024-08-02

**Authors:** Alsadig Suliman, Hussein Elfaki, Sara Hussein

**Affiliations:** Department of General Surgery, Sudan Medical Specialization Board, Khartoum, Khartoum, Sudan

**Keywords:** Amyand's hernia, Case report, Sudan

## Abstract

**Introduction and importance:**

Amyand's hernia (AH) is an extremely rare type of inguinal hernia where the vermiform appendix is present within the inguinal hernia sac. This report documents the first known instance of AH in Sudan, highlighting its unprecedented occurrence in this region.

**Case presentation:**

A 2-month-old infant presented to the ER with an obstructed right-sided inguinal hernia for 6 h. During surgical repair, an inflamed appendix was found within the hernial sac. The appendix was ligated, excised, herniotomy was performed to repair the hernia, and the appendix was sent for histopathological examination.

**Clinical discussion:**

This case underscores the importance of recognizing rare presentations of common conditions in different geographical and demographic contexts. The co-occurrence of acute appendicitis and AH in infants poses a diagnostic challenge, often only detected incidentally during surgical exploration.

**Conclusion:**

By presenting this unique case, we aim to raise awareness about the potential for such rare hernias in pediatric populations. Early recognition and proper management are crucial for ensuring optimal patient outcomes and preventing complications associated with delayed diagnosis or treatment.

## Highlights

• First documented case of Amyand's hernia (AH) in Sudan.
• AH diagnosed in a 2-month-old infant with an obstructed right-sided inguinal hernia.
• Surgical repair revealed an inflamed appendix within the hernial sac.
• Case emphasizes the need for early recognition and management of rare hernias in infants.
• Raises awareness of AH in pediatric populations in diverse geographic regions

## 1 Introduction

An inguinal hernia is the protrusion or passage of the peritoneal sac, with or without abdominal contents, through a weakened part of the abdominal wall in the groin. This occurs when the peritoneal sac enters the inguinal canal either indirectly through the deep inguinal ring or directly through the posterior wall of the inguinal canal [1]. Inguinal hernias are common in infants and frequently require surgical repair [2]. Amyand's hernia (AH), defined as the presence of an appendix within an inguinal hernia sac, was first described by Claudius Amyand in 1735 [3]. This rare condition accounts for only 1 % of inguinal hernia cases, with acute appendicitis within the hernial sac occurring in only 0.08 % of cases. Male infants are more likely to experience this condition, possibly due to their patent processus vaginalis [4,5,6]. A definitive preoperative diagnosis of AH is challenging due to nonspecific clinical signs and the lack of specific radiological features. Only 182 pediatric cases have been reported in the past 20 years [6,7]. The rarity of documented cases in certain regions, such as Sudan, adds to the clinical and academic value of reporting such instances. This case report describes a rare instance of AH in an infant, detailing the clinical presentation, diagnostic approach, surgical intervention, and postoperative management. This manuscript was reported in line with the SCARE criteria [8].

## 2 Case presentation

**Fig. 1:**
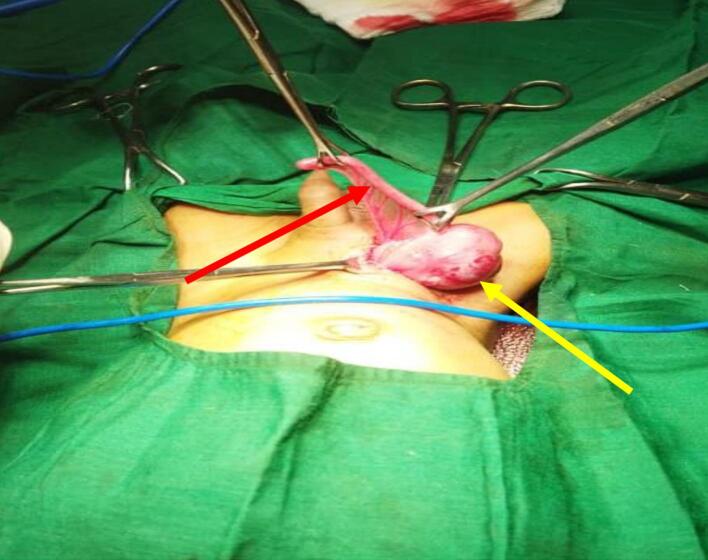
Intraoperative image of AH. Red arrow: inflamed appendix. Yellow arrow: healthy cecum. (For interpretation of the references to colour in this figure legend, the reader is referred to the web version of this article.)

A 2-month-old male infant was brought to the ER with a 6-hour history of irreducible swelling in the right inguinal region, abdominal distension, vigorous crying, and two episodes of bilious vomiting. Examination revealed an irritable infant with no dysmorphic features. Vital signs were PR: 140 bpm, RR: 39 cpm, Temperature: 37.3 °C, and weight 3.7 kg. The abdomen was distended but soft, with no tenderness, guarding, or rigidity. A prominent, irreducible bulge measuring 4 × 3 cm was noted in the right inguinoscrotal region, with the right testis palpable in the upper scrotum. Due to the clear clinical diagnosis, an ultrasound examination was not pursued. Blood tests revealed a significant elevation in the white blood cell count (11,300/ml). An emergency inguinal exploration through a right transverse inguinal incision revealed the presence of the caecum and inflamed appendix inside the hernial sac, leading to an intraoperative diagnosis of AH [Fig. 1]. There was no macroscopic evidence of appendix perforation, and no abdominal contamination was noted. The caecum appeared normal, indicating simple uncomplicated appendicitis. The inflamed appendix was carefully dissected from the hernia sac, with the appendiceal base identified and the mesoappendix ligated. The appendix was excised, and the hernial sac was securely ligated and divided. The appendix was sent for histopathology, which indicated reactive hyperplasia of lymphoid tissue, suggesting acute inflammation. The postoperative recovery was uneventful, and the patient was discharged on the third postoperative day. Follow-up visits showed no complications.

## 3 Discussion

The appendix, when present inside the hernial sac, may develop adhesions and become more susceptible to inflammation compared to a normal anatomical appendix. These adhesions play a significant role in keeping the appendix in this location, making it more superficial and vulnerable to trauma [9,10]. The appendix inside the hernial sac may be just incarcerated and entirely healthy, inflamed, or, in some cases, perforated. Several theories have been proposed to explain the pathophysiology of AH, but the cause-effect relationship between inguinal herniation and appendicitis remains unclear. It is debated whether appendicitis is the primary pathological mechanism or if the primary event is herniation of the appendix. Muscular spasms may compress the appendix, reducing blood flow, and promoting inflammation and bacterial development [6,9,11].

Diagnosing AH preoperatively remains challenging due to its nonspecific clinical presentation. Out of 60 cases reviewed over 12 years, only one had a preoperative diagnosis [9]. Standard diagnostic tools for inguinal hernias include physical examination and imaging studies. In this case, an ultrasound was skipped due to urgent symptoms resembling an obstructed hernia, requiring immediate intervention within the critical golden hour. However, in general practice, imaging studies such as ultrasound and CT scans can provide valuable information. Ultrasound is particularly useful for identifying the contents of the hernia sac and assessing vascularity, which can help in differentiating AH from other types of inguinal hernias. CT scans, though less commonly used in infants due to radiation exposure, can offer detailed anatomical information and are beneficial in complex cases or when the diagnosis is uncertain [10].

The inguinal approach is the gold standard for treating AH [6] although laparotomy may be necessary in complicated cases. In addition, laparoscopic surgery can be technically demanding in infants, but it offers advantages such as minimal invasiveness, better visualization of the deep inguinal ring on both sides, reduction of irreducible contents, improved assessment of the vasculature, and faster recovery [5].

When the appendix within the hernia sac is inflamed, performing an appendectomy is crucial to prevent complications such as perforation and peritonitis [12]. A normal appendix in the hernia sac does not necessitate appendectomy unless it is affected by other issues [4]. Other less common indications for appendectomy include the deformation of the appendix and its dense adhesions to the hernial sac [5]. If the cecum is also affected, a more extensive procedure, such as a right hemicolectomy, may be necessary if necrosis is present [7]. Hernia ligation is performed in all cases of AH, regardless of the appendix's condition, with high ligation of the processus vaginalis being the standard approach [5].

In this case report, the patient underwent an inguinal approach with appendectomy due to an inflamed appendix within the hernia sac. The cecum was healthy and simply reduced into the abdomen. The procedure was successful, with no postoperative complications noted during follow-up.

One of the key strengths of this report is its documentation of the first known case of AH in Sudan, adding valuable data to the medical literature and helping to broaden the understanding of this rare condition in different geographic regions. The detailed account of the clinical presentation, surgical intervention, and postoperative management provides a comprehensive overview that can aid clinicians in recognizing and managing similar cases.

However, the report also has limitations. Given that this is a single case study, the findings may not be generalizable to all populations or settings. All authors attest that they meet the current ICMJE criteria for Authorship.

## 4 Conclusion

This case report highlights the importance of early recognition and prompt surgical management for positive outcomes in cases of AH. By increasing awareness of this condition in pediatric patients, it underscores the necessity of considering AH in the differential diagnosis of irreducible inguinal hernias. The report also emphasizes the significance of documenting rare cases from diverse geographical regions to enhance the global understanding of such medical conditions.

During the preparation of this work, the authors used ChatGPT in order to improve language and readability with caution. After using this tool, the authors reviewed and edited the content as needed and take full responsibility for the content of the publication.

## Informed consent

Informed consent was obtained from the patient's parents.

## Ethical approval

This case report did not require ethics approval as it involves a single patient case that does not constitute research according to our institution's guidelines. The Institutional Review Board (IRB) of Sudan Medical specialization board has confirmed that ethical approval is not necessary for case reports of this nature.

## Funding

No extramural funds were used to support this case report.

## Author contribution

Alsadig Suliman: Conceived and designed the study, drafted the introduction and discussion sections, and reviewed the manuscript critically for important intellectual content.

Hussain Alfaki: Gathered and analyzed the patient data, drafted the case presentation section.

Sara Hussain: contributed to drafting the abstract and conclusion sections also contribute to design and soft work.

## Guarantor

Alsadig Suliman.

## Research registration number

Not applicable.

## Conflict of interest statement

The authors declare no conflicts of interest. The authors confirm the accuracy of the data.
